# Regulatory complexity of cellular differentiation in *Candida albicans* revealed through systematic screening of protein kinase mutants

**DOI:** 10.1128/mbio.01698-24

**Published:** 2024-07-26

**Authors:** Michael C. Lorenz

**Affiliations:** 1 Department of Microbiology and Molecular Genetics, University of Texas McGovern Medical School, Houston, Texas, USA; Tel Aviv University, Tel Aviv, Israel

**Keywords:** *Candida albicans*, hyphal development, protein kinases, mutant libraries

## Abstract

A recent study in *mBio* reports the construction and preliminary screening of a library containing mutants of 99 of the 119 predicted protein kinases in *Candida albicans* (the majority of the remaining 20 are probably essential) (J. Kramara, M.-J. Kim, T. L. Ollinger, L. C. Ristow, et al., mBio e01249-24, 2024, https://doi.org/10.1128/mbio.01249-24). Using a quantitative competition assay in 10 conditions that represent nutritional, osmotic, cell wall, and pH stresses that are considered to model various aspects of the host environment allowed them to phenotypically cluster kinases, which highlight both the integration and specialization of signaling pathways, suggesting novel functions for many kinases. In addition, they tackle two complex and partially overlapping differentiation events, hyphal morphogenesis and biofilm formation. They find that a remarkable 88% of the viable kinase mutants in *C. albicans* affect hyphal growth, illustrating how integrated morphogenesis is in the overall biology of this organism, and begin to dissect the regulatory relationships that control this key virulence trait.

## COMMENTARY

The use of systematically constructed genome-scale ordered mutant libraries to probe gene function was pioneered with the 1999 publication of a set of just over 2,000 mutants in *Saccharomyces cerevisiae* (1). The application of this concept to fungal pathogens was slowed by limited genetic tools but, as these have improved, mutant libraries have been developed for several species, including *Candida albicans* (2), *Cryptococcus neoformans* (3), and *Aspergillus fumigatus* (4). The construction of these libraries remains laborious, but they have provided important insights into virulence mechanisms in these and other species. Krysan et al. recently reported the construction and analysis of a targeted but comprehensive library containing mutants in essentially all non-essential protein kinases in *C. albicans* (5).

Their primary screen was a competition experiment in which the mutant strains were mixed with a fluorescently marked control and grown together for multiple generations. This is a more sensitive way to identify fitness differences than the typical monoculture approach and closer to a natural evolutionary scenario, where a mutant arises in a population of nearly isogenic cells. They use flow cytometry to determine the exact proportion of each strain, allowing the calculation of a quantitative Fitness Score for each mutant in each condition, which was then used to generate a phenotypic heat map to cluster mutants based on shared behaviors. In many cases, such as the Hog (high osmolarity glycerol), Mkc (cell wall integrity), and Snf (sucrose non-fermenting) pathways, the known kinase components clustered together. In others (Cek MAPK, Cka casein kinase, and Tpk protein kinase A), they did not. The authors propose that this reflects limitations in their methodology, a reasonable caveat. Yet, they also recognize that the lack of concordance may reflect an unappreciated complexity in these regulatory structures. Indeed, their data suggests this to be the case for several pathways.

Two additional sets of screens for phenotypes core to *C. albicans* biology, hyphal growth and biofilm formation, were not included in the phenotype heat map because the assays are more qualitative in nature. The ability of *C. albicans* to transition between yeast and hyphal morphologies is critical to virulence in this species and a vast array of stimuli promote this differentiation (6). Here, six inducing conditions were tested and a staggering 88 of the 99 mutants showed phenotypes in at least one of them; 30 were affected in all six conditions (and 12 of these had not been previously reported to have hyphal defects). Even though morphogenesis is central to fungal biology (and the collective mind of the *Candida* research community), that all but 11 kinases alter hyphal phenotypes is remarkable.

Biofilm formation is key to many manifestations of *Candida* infections and its regulation is complex (7). When screened in tissue culture medium and Spider medium, mutation of 33 of the kinases negatively impacted biofilm formation. A very significant number to be sure, but far fewer than had hyphal phenotypes, which is interesting given the overlap in the transcriptional regulation of these two differentiation events. One caveat is that they screened the full library in only these two conditions, but this is still roughly half the number of mutants that showed hyphal phenotypes in any random selection of two of their hyphal growth conditions, suggesting that biofilm formation is a subset of the hyphal program.

Several key takeaways come from this work. First, context is everything. Only two mutants had phenotypes in all 10 environmental conditions, while 15 mutants affected hyphal growth in only one of the six media. Deletion of *MKC1*, encoding a kinase in the cell wall integrity pathway, affected hyphal growth *in vitro*, but not in a mouse ear tissue infection model that allows intravital imaging; the authors previously showed discrepencies between *in vitro* and *in vivo* hyphal phenotypes of mutants of *TPK1* and *TPK2*, which encode protein kinase A, using the same model (8). Second, the environmental contingency of kinase phenotypes may explain often conflicting literature; *hog1*∆ mutants have been reported to either have no hyphal phenotypes or to be hyperfilamentous and here they find that Hog1 promotes, represses, or has no role depending on the conditions. Further, while drawing tidy circuits to represent signaling pathways (MAPKKK→MAPKK→MAPK) is satisfying, it has been clear for some time that few pathways are so linear. For example, mutants of the Bck1 MAPKKK were hypofilamentous in all conditions tested, while the downstream Mkk1 and Mkc1 had more restricted phenotypes, suggesting that Bck1 transduces signals to proteins other than those in its canonical pathway. Similarly, overexpression of the Hgc1 cyclin bypasses the filamentation block in the supposedly downstream *cbk1*∆ mutant.

The development of mutant libraries in *C. albicans* was complicated by limiting methodologies and the diploidy of this species. Progress was made thanks to a combination of genetic cleverness and brute force, the details of which can be found in a recent review (9). The two mutant libraries that have seen the most use are the Gene Replacement and Conditional Expression (GRACE) library, originally constructed by Roemer and Bussey and consisting of 1,100+ strains (later expanded to more than 2,300) in which the only functional copy of a given gene is under the control of a doxycycline-repressible promoter (10, 11), and a transcription factor knockout library created by Homann and Johnson (2). Two earlier efforts to construct and screen protein kinase libraries should be mentioned, one from the Mitchell lab that included 80 mutants used to identify cell wall regulators (12) and another from Morschhauser et al. initially screened for metabolic regulators (13).

It is the Homann transcription factor library that Kramara et al. have intentionally to emulated, including using the same set of bar codes (signature tags) that permit mutants to be tested in pools, such as in *in vivo* experiments. The transcription factor and kinase libraries share several advantages: they are limited in number, making it easier to screen in multiple conditions in multiple conditions, and are “target-rich,” meaning that they are more likely to show phenotypes than individual knockouts of the downstream effectors they regulate. The question of whether to prefer screens of large libraries or more targeted smaller ones is philosophical, but the present work suggests opting for smaller libraries can be very informative; indeed, Kramara, Krysan and colleagues have given us a mountain of data to consider and a very useful community resource.
